# The Challenges of Pain Assessment in Geriatric Patients With Dementia: A Review

**DOI:** 10.7759/cureus.49639

**Published:** 2023-11-29

**Authors:** Salah N El-Tallawy, Rania S Ahmed, Shamah M Shabi, Fatoon Z Al-Zabidi, Abdul Rehman Z Zaidi, Giustino Varrassi, Joseph V Pergolizzi, Jo Ann K LeQuang, Antonella Paladini

**Affiliations:** 1 Department of Anesthesia and Pain Management, College of Medicine, King Saud University, Riyadh, SAU; 2 Department of Anesthesia and Pain Management, Faculty of Medicine, Minia University, Minia, EGY; 3 Department of Anesthesia and Pain Management, Faculty of Medicine, National Cancer Institute (NCI) Cairo University, Giza, EGY; 4 Department of Family Medicine, College of Medicine, Alfaisal University, Riyadh, SAU; 5 Department of Pain Medicine, Paolo Procacci Foundation, Rome, ITA; 6 Department of Research and Development, NEMA Research, Inc., Naples, USA; 7 Department of Life, Health and Environmental Sciences (MESVA), University of L'Aquila, L'Aquila, ITA

**Keywords:** "pain management with dementia", "old age", "geriatric", "pain assessments using recent technology", "causes of pain with dementia", "pain assessment with dementia", "dementia"

## Abstract

Pain in dementia patients is common, poorly measured, and undertreated. It is important to discuss the challenges in the pain assessment and management to find a possible solution for adequate pain management. The aim of this article is to discuss the challenges in the assessment of pain in geriatric patients with dementia. An extensive online database search was conducted via multiple websites using the following keywords: "dementia," "pain assessments," "pain assessment with dementia," "causes of pain with dementia," "pain assessments using recent technology," "geriatric," and "old age" to identify the relevant articles. Our inclusion criteria were articles that focused on pain in geriatric patients diagnosed with dementia, in English, published between January 2018 and January 2023, and available as free full text and those which were clinical trials, observational studies, review articles, systemic reviews, meta-analysis, or case series. The exclusion criteria were articles that did not have pain in geriatric patients diagnosed with dementia as their primary focus, involving geriatric or non-geriatric patients with major psychological distress, not in the English language, not published between January 2018 and January 2023, and not available as free full-text and those which were case reports and editorial articles. After manually excluding the articles that did not meet our inclusion criteria, we ended up with 38 articles. In conclusion, any instruments have been made for the pain assessment in patients with dementia. The two most common tools used to assess pain in this vulnerable population are the Pain Assessment in Advanced Dementia (PAINAD) and Pain Assessment Checklist for Seniors with Limited Ability to Communicate (PACSLAC) scales. The utilization of new technology may offer promising solutions for the pain assessment in patients with dementia.

## Introduction and background

Dementia is an acquired disease that causes a decline in cognitive function, and it is responsible for a decline in memory, attention, language, social cognition, executive, and motor function [1]. More than 55 million people globally are diagnosed with dementia, most of whom are in low- or middle-income countries [2]. There are many causes of dementia, but the two most common causes are Alzheimer's disease and vascular diseases [3]. Regardless of the pathophysiology, all of them generate a decline in cognitive function with loss of short-term memory as the first sign. Dementia is a disease of the elderly; hence, aging is an important risk factor for dementia. Other risk factors include smoking, obesity, and uncontrolled diabetes [2,3]. There is no cure for dementia, and the management is symptomatic [3]. In people with dementia, pain is a crucial issue that deserves to be discussed. Not only do the elderly experience pain often; dementia patients experience pain than the general population [4,5]. Yet, because of their limited ability to communicate, their pain is often underestimated and undertreated. Many elderly patients are prescribed antipsychotics and antidepressants when their underlying condition may actually be pain [4,5]. The assessment of pain in geriatric patients with dementia is a complex task for healthcare professionals. Due to the cognitive and communicative impairments associated with dementia, assessing pain in these patients is often challenging. Many tools for the assessment of pain have been developed such as the Pain Assessment in Advanced Dementia (PAINAD) and Pain Assessment Checklist for Seniors with Limited Ability to Communicate (PACSLAC); however, they mostly rely on observing the behavior of the patient [6]. The aim of this review is to highlight the challenges of pain assessment in geriatric patients with dementia.

## Review

Methods

An extensive computer search was done using databases including PubMed, the Cambridge Structural Database, the Cochrane Library, Embase, Medscape, the National Center for Biochemistry Information (NCBI), and the World Health Organization (WHO). The search strategy included related keywords such as "dementia," "pain assessments," "pain assessment with dementia," "causes of pain with dementia," "pain assessments using recent technology," "geriatric," and "old age." Both the titles and abstracts of the identified articles were screened by two independent reviewers to identify which research studies were eligible for inclusion in the review article.

**Figure 1:**
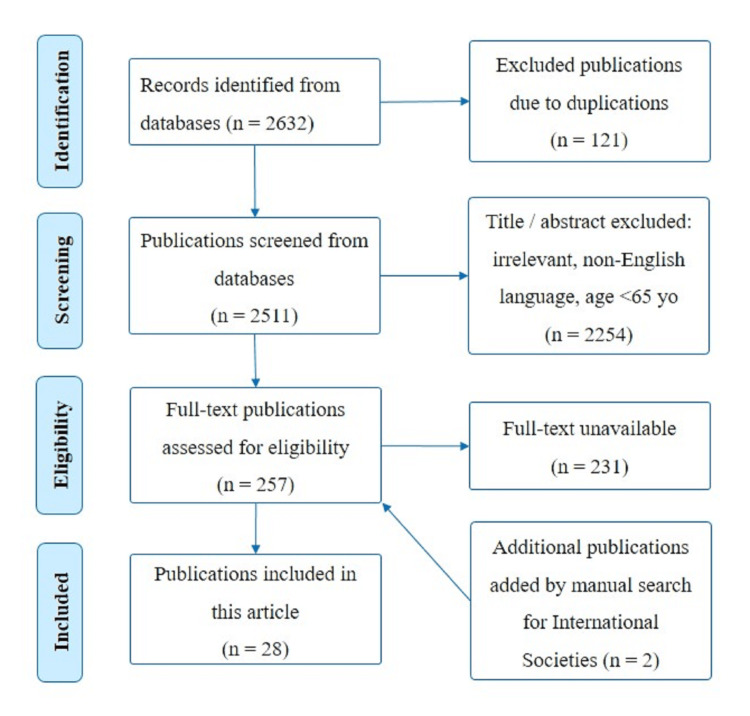
Flowchart of the included articles

The inclusion criteria included articles published in the English language between the years 2018 and 2023, articles that were available in free full text, articles focusing on geriatric individuals diagnosed with dementia, articles involving human subjects, clinical trials, observational studies, review articles, or systematic reviews and meta-analysis. Exclusion criteria were articles published in non-English language, articles that were not available in free full text, articles involving geriatric or non-geriatric patients with major psychological distress or major systemic illnesses, case reports, and editorial articles. The final articles that met the inclusion criteria (n=28) and included in this review are shown in Figure 1.

Definitions

Pain is defined by the International Association for the Study of Pain as "an unpleasant sensory and/or emotional experience associated with, or resembling that associated with, actual or potential tissue damage." Inadequate verbal communication does not rule out the potential that a person is in pain and needs to be offered adequate pain-relieving treatment [7].

Geriatric patients are elderly patients. The WHO defines anyone who is 65 years or older as a geriatric patient [8]. Dementia, also known as a major neurocognitive disorder, is a term used to describe several diseases that affect the cognitive function of the brain. According to the Diagnostic and Statistical Manual of Mental Disorders (DSM-5), dementia is an acquired disorder of cognitive function that is commonly characterized by impairments in memory, attention, language, executive function, social cognition, and/or motor domains [1].

Dementia

Prevalence of Dementia

Dementia is mostly considered a disease of the elderly. More than 55 million people globally are diagnosed with dementia [2]. More than 60% of those people are in low- or middle-income countries. According to statistics, there are around 10 million more cases of dementia diagnosed each year [2]. It is expected that the number of people affected by dementia will double by the year 2050 [9].

Etiology of Dementia

There are a lot of causes for dementia. Some of these causes include Alzheimer's disease which is responsible for about 70% of the cases of dementia and vascular dementia which is responsible for about 20% of the cases of dementia, while the remaining cases may be associated with Lewy body dementia and less commonly neurodegenerative dementia diseases like Parkinson's disease, Huntington's disease, frontotemporal lobe dementia, and prion disease [3].

Risk Factors of Dementia

Aging is the most important risk factor for dementia. Other risk factors include a family history of dementia, uncontrolled hypertension, smoking, obesity, uncontrolled diabetes, excessive alcohol consumption, low physical activity, social isolation, and depression [2,3].

Clinical Features of Dementia

The symptoms of dementia might vary widely depending on the cause of dementia. Some symptoms may worsen over time; others might appear only in the beginning or late stages of the disease. But generally, short-term memory loss and forgetfulness are the first signs of dementia. Other early indications of dementia include getting lost while driving or walking, being confused in familiar settings, losing track of time, having trouble following conversations or finding words, difficulty performing routine tasks, and visually misjudging distances to objects. Dementia also affects the mood of the patient such that they might feel anxious, sad, or agitated regarding their memory loss, have changes in personality and behavior, be socially isolated, and lose interest in other people's emotions [2,3,10].

Diagnosis of Dementia

To diagnose someone with dementia, a full examination needs to be carried out starting by taking a detailed history to look for any cognitive decline, evaluating for any risk factors, looking for any of the signs and symptoms specific to dementia, and performing a complete physical examination, full neurological and mental status examinations, and cognitive function test [3,9,11]. The mental status examination describes the patient's mental status and behavior both qualitatively and quantitatively at a specific time [9,11]. It consists of five components assessing the general appearance and behavior, cognition, mood, speech, thought process, thought content, perceptual disturbances, and insight and judgment. However, this is a very lengthy exam that is preferentially performed when the patient is highly suspected to have dementia. Another test that most physicians use to screen for dementia and assess cognition is the Mini-Mental State Examination (MMSE) since it is shorter [12]. MMSE consists of 11 questions and tests for orientation to time and place, attention, short-term memory, language skills, visual and spatial relationships between objects, and the ability to understand and follow instructions. There are 30 items, and a score <24 is suggestive of cognitive impairment [12,13]. It is also important to exclude any reversible causes for cognitive impairment such as delirium, major depressive disorder, and any medications that the patient might be taking. Some lab tests that are ordered include vitamin B12 to assess for deficiency, thyroid-stimulating hormone (TSH) to assess for hypothyroidism, complete blood count (CBC) to look for anemia, comprehensive metabolic profile to assess for any kidney or liver diseases and hypo- or hyperglycemia, urinalysis to rule out infection, and a urine toxicology screening, although it is less likely for an elderly person to take recreational drugs. A brain magnetic resonance image (MRI) might be ordered to find the underlying cause of dementia. Finally, to confirm the diagnosis of dementia, physicians use DSM diagnostic criteria for neurocognitive disorders and classify the patient based on the severity of dementia [9,11].

Management of Dementia

Unfortunately, there is no cure for dementia. However, the first step is to identify the possible underlying causes of dementia and manage them accordingly, because each cause has a different approach. In general, the management of dementia requires a multidisciplinary approach [3]. Pharmacological treatment of dementia includes medications that can slow down cognitive decline and decrease sleep disorders, depression, anxiety, agitation, and psychosis [3,9,14,15]. Supportive management for patients with dementia includes maintaining a healthy lifestyle by staying active, eating healthfully, and avoiding smoking and drinking alcohol; it is also beneficial having a routine and staying in a familiar environment [1,14,15]. An occupational therapist and speech therapist might be involved in the later stages of dementia to find ways to carry out daily activities easily and help with swallowing and speaking difficulties [3].

Pain in Dementia Patients

Pain is very common in people with dementia [6]. It is estimated that 50% of older people and 80-85% of older people with dementia suffer pain as a result of various chronic conditions [16]. Pain management is an important health issue and can minimize the negative impacts on the quality of life for those patients. Pain can lead to difficult behaviors, such as sadness, anxiety, and decline in physical functions. Due to neuropathological changes in demented patients, the perception of pain will even change, making it even more challenging to recognize and keep track of pain in dementia patients due to progressively reduced cognitive and verbal abilities [4]. In addition, pain can result in changes to the endocrine, metabolic, gastrointestinal, hepatic, cardiovascular, and central nervous systems [17].

Challenges of Pain Assessment and Management in Geriatric Patients with Dementia

Pain is a subjective symptom and is difficult to assess because everyone has different pain tolerances. Several instruments have been developed to assess pain [18]. Pain in patients with dementia is often overlooked, underestimated, and undertreated, because patients cannot expressed their needs clearly [19]. Despite efforts to better recognize pain in this vulnerable population, pain is still not well assessed, diagnosed, or controlled [5,16]. The reasons for poor pain assessment and management involve communication barriers, limited expertise of healthcare workers, misconceptions, particularly regarding the pharmacological treatment of pain, and misinterpretation of patient behaviors [5,20]. The accuracy of pain evaluation in older, cognitively impaired persons is also uncertain, according to prior studies, which makes healthcare practitioners hesitant to administer analgesics [5,6]. Also, clinicians' educational backgrounds vary, which may contribute to variations in how patients' behaviors are interpreted [5]. Other barriers to pain management in people with dementia are diagnostic mistakes and a lack of validated tools for pain assessment [17]. Moreover, many patients with dementia are given psycholeptics and antidepressants instead of analgesic medications, because nurses might misinterpret an expression of pain as hallucinations and agitation, which may accompany dementia. In fact, nursing home personnel may find it difficult to recognize pain-related behaviors and thus underestimate the patients' pain levels and need for analgesics [5].

The question about appropriate analgesics can be a difficult one. Older adults with dementia who live in communities, receive home care, or are institutionalized tend to use opioids less frequently compared to those without dementia, according to earlier studies [21]. Nonetheless, some older adults with dementia take opioids at a frequency that is equivalent to or higher than the general population without considering the severity of the patients' pain. The absence of pain indicates effective pain management. Prior studies showed no significant differences between acetaminophen and placebo in patients with low back pain or osteoarthritis in terms of pain, disability, or quality of life. The nonsteroidal anti-inflammatory drugs (NSAIDs) are not recommended in this age group because of the associated side effects on the cardiovascular system, renal impairment, and the increased risk of gastrointestinal bleeding [22].

Pain Assessment Tools

Patient self-report scales are the gold standard for pain assessment in the general population; patients may be asked to rate their pain verbally, numerically, or visually [23]. These tools work well in patients who are able to verbally communicate their pain, including patients with mild to moderate dementia. However, self-report scales may be not appropriate for patients with severe dementia with limited ability to communicate. As a result, different methods have been suggested in order to assess pain in patients with dementia, which can be used by nursing staff in nursing homes, clinics, and hospitals [5,20]. Other researchers have used observational reports (usually the caregivers) to gather data about pain and other aspects of health in people with dementia. Accordingly, the pain assessments have focused on two main approaches: behavioral observations and caregiver reports [20]. These tools are based on the observation of pain-related actions, vocalizations, and facial expressions. Although these measurement tools have undergone extensive development, they frequently fail to provide sufficient evidence of psychometric properties (such as reliability, face and construct validity, responsiveness, and usability), and they are not widely used internationally [4]. Some of the commonly used tools for the assessment of pain are the Abbey Pain Scale, Comprehensive Pain Assessment, Doloplus-2 Pain Assessment Scale, Non-Communicative Patient's Pain Assessment Instrument, Checklist of Nonverbal Pain Indicators, PACSLAC, Automatic Pain Assessment with Video Systems, and PAINAD [6,24,25].

PAINAD: The PAINAD scale is generally recognized as the most appropriate method for evaluating pain in clinical settings, while the PACSLAC scale is the preferred method for research sittings [6]. The psychometric characteristics and simplicity of the PAINAD scale make it an easy-to-use tool for the assessment of pain in patients with communication impairment. The PAINAD scale is designed to evaluate the following parameters: breathing, negative vocalization, facial expression, body language, and the ability of the patient to be consoled [24]. The PAINAD scale requires that the health practitioners have proper training before its use. It has been verified and culturally adapted for use in several nations, including Singapore, Germany, the Netherlands, Italy, China, the United Kingdom, Brazil, Turkey, and the United States [6].

PACSLAC: The PACSLAC scale assesses pain by looking at pain-related behaviors, looking at the "facial expressions, activity, body movements, social and personality mood, and others including physiological data and specific pain vocalization." This scale has strong internal consistency, good test-retest reliability, and the ability to distinguish between painful conditions and nonpainful ones. This scale is valid and reliable for both clinical uses and research [25].

High-technology tools for pain assessments

Artificial Intelligence (AI)

Several new technologies such as AI have been described to help in pain assessment and make it more reliable, especially in patients with moderate to severe dementia. The new technology methods depend on many tools such as facial expressions and facial muscle movements, vocal cord responses, and behavioral changes due to pain. The Automatic Pain Assessment with Video Systems can be used for elderly patients with dementia and may be used with the other methods for pain assessments. The tool focuses mainly on the automatic analysis of facial expressions [20,26,27].

Smart Wearable Shirts

Another tool for pain assessment is the use of smart wearable shirts enabling the continuous monitoring of human physiological signs, including heart rate, respiratory function, and changes in regular respiration, body movements, and pacing, without any disturbance to the activities of daily living [20,28].

Smart Home

A smart home is a residence equipped with internet-connected devices. They are wireless devices and can be used to collect, transfer, store, and analyze data over a network. These tools may be more accurate and minimize the reporting biases found in other clinical methods [20,29]. These technologies can help improve the reliability of pain assessment tools. Machine learning algorithms and data analysis techniques can be used to assess pain by looking at the changing of patient's facial expressions and analyzing their vocal cord sounds [27].

Management of pain with dementia

Pain management in elderly patients can be achieved by non-pharmacological and pharmacological treatments.

Non-pharmacological Treatment

Non-pharmacological treatment includes physical therapy, physical exercise, massages, heat or cold therapy, and transcutaneous electrical nerve stimulation (TENS). Alternative therapies include acupuncture, cupping, and aromatherapy. Other types of non-pharmacological treatment in elderly patients include educational programs and psychological methods which can be used in selected patients [30,31].

A systematic review recommended that behavioral therapies targeting pain and pain interventions targeting behavior may both be useful in lowering pain and reducing behavioral symptoms in dementia patients. Leisure and recreation may also reduce pain. The majority of leisure activities are entertaining, are emotionally stimulating, and have a positive impact on people with dementia [32]. Patients who have cognitive disabilities but who pursue happiness on their own or through social interaction with other residents can benefit from recreational activities to lift their spirits. For nursing home residents, leisure activities promote quality of life, including enjoyment, purposeful activities, functional capacity, and socialization. Clinical studies showed that the play activities program (PAP) helped frail older persons in chronic pain by reducing their pain and improving their functional mobility and mental health. The result of this study demonstrated the effectiveness of an eight-week PAP for dementia patients residing in nursing homes in easing their discomfort and enhancing their psychological well-being. They suggested that including play activities in routine care could be advantageous for residents of nursing homes and the people who provide care for them [16,32].

A study published in 2020 assessed the impact of pain management for nursing home patients with dementia using interaction with a robotic seal named PARO. PARO demonstrated the potential in lowering pain and drug use for those with chronic pain. As an alternative to traditional methods of pain management for patients with dementia, this intervention might be introduced into routine care. Care providers must weigh the advantages and drawbacks of integrating social robots into clinical practice while also taking the residents' personal preferences into account. The utility of PARO in long-term care settings has to be tested further in larger, longer-term randomized controlled trials [33].

Pharmacological Treatment

Pain management in elderly patients is complex and multifactorial due to the associated cognitive disorders, comorbidities, multi-organ dysfunction, and polypharmacy [34]. The aging process is associated with changes in safety, toxicity, pharmacokinetic, and pharmacodynamic characteristics of the medications, and this should be considered in any treatment program. The treatment outcome could be influenced by the degree of cognitive impairments. Some patients may receive sedative, antipsychotic, and anti-epileptic medications, all of which can interact with the pain medications. All these factors should be taken into consideration when choosing pain medications.

According to the British Geriatrics Society guidelines, the recommended treatment depends on the severity and type of pain. Pain medications should be tested slowly and titrated gradually [30,31]. It is good to remember the old clinical saying "Start low and go slow." The pain medications include non-opioid analgesics, opioids, and adjuvant drugs. It is recommended not to start with opioids, although opioids are needed to manage severe pain. Pain control in elderly patients with dementia by the use of psychoactive drugs (e.g., neuroleptics and benzodiazepines) and anti-convulsants (e.g., gabapentinoids) should be avoided or used with caution, because these combinations may worsen the clinical condition of the patients [30].

## Conclusions

Assessment of pain in elderly patients with dementia is very complex and challenging and requires training and good observation skills. Different methods for the assessment of pain with dementia have been developed, and the two most common tools used for pain assessment in this vulnerable group are the PAINAD and PACSLAC scales. However, both scales are based on observing the behavior of the patient which can lead to variable measurements depending on the expertise of the clinicians and how well they know the patient. Recently, since we are in the era of technology, the utilization of AI may offer promising solutions for the pain assessment in this group of patients. These smart tools may minimize the reporting biases found in other clinical methods. As a result, future research is encouraged to develop more reliable pain assessment instruments for patients who are not able to communicate their pain.
